# Multi-omics insights into the microbial and metabolic drivers of regional flavor diversity in Guizhou traditional fermented fish

**DOI:** 10.3389/fmicb.2026.1821104

**Published:** 2026-06-18

**Authors:** Anqin Zhu, YanLi Wang, Jin Zhang, Shiping Lu, Chuanbo Zhang

**Affiliations:** 1 Laboratory of Microbial Resources and Industrial Application, College of Life Sciences, Guizhou Normal University, Guiyang, China; 2 Department of Agronomic Engineering, Guizhou Vocational College of Agriculture, Guiyang, Guizhou, China; 3 Huangping Yedonghe Yuan Ecological Recycling Farming Co., Ltd., Guiyang, China

**Keywords:** fermented fish, flavor diversity, metabolome, microbial diversity, multi-omics

## Abstract

The flavor quality of traditional fermented fish in Guizhou exhibits significant regional variations, yet its microbial and metabolic mechanisms remain unclear. This study integrated sensory evaluation, high-throughput sequencing, and untargeted metabolomics to compare the quality, microbial communities, and metabolite profiles of fermented fish from Jinping, Liping, and Tianzhu. Results showed that Liping samples demonstrated optimal performance in flavor harmony, acidity perception, and texture. Tianzhu samples exhibited the lowest microbial α-diversity, with *Staphylococcus* accounting for over 99% of bacterial communities, whereas Jinping and Liping samples were enriched with *Weissella*, *Saccharomyces*, and *Debaryomyces*. Metabolomic analysis identified a total of 1,372 metabolites. PLS-DA revealed significant separation of metabolic profiles among the three groups, with differential metabolites primarily enriched in amino acid metabolism and the citric acid cycle. Liping samples exhibited significant upregulation of L-glutamine, citric acid, and L-glutamate. Spearman correlation analysis indicated that *Weissella* and *Saccharomyces* were positively correlated with the aforementioned flavor metabolites. This multi-omics study demonstrates that specific microbial community structures and their metabolic activities are core factors driving regional flavor differences in Guizhou traditional fermented fish, providing a theoretical basis for improving product quality through targeted microbial community regulation.

## 1 Introduction

Fermented fish is one of the most valued traditional, naturally fermented foods in Miao and Dong communities, boasting a unique flavor, delicious taste, and high nutritional value. The fermented fish, prepared by frying, roasting, or steaming, is considered a premium dish and is served to honor guests (Zhang et al., 2023). In September every year, fresh fish (*Cyprinus carpio L.*) are harvested from rice fields for producing fermented fish. Briefly, the gill and internal organs were removed, the treated fish were marinated in 15% salt for 24 h, supplemented with a small amount of high concentration Chinese liquor, and marinated for an additional 1–2 d. The marinated fish were stuffed with a mixture of fermented rice (JiuPei), steamed glutinous rice, red yeast rice (fermented with *Monascus purpureus*), chili, ginger, star anise, pepper, and other spices, etc. The stuffed fish were then layered into a Chinese fir bucket, covered with lotus leaves, weighed down with pebbles, and stored in a shaded place (25–26 °C) (Sun et al., 2022). The fermentation was completed after 3 or 4 months. The resulting fermented fish had a ruddy color, a sour and salty taste, and a fragrant aroma (Sun et al., 2022).

Relying on natural fermentation, traditional fermented fish products across China exhibit distinct quality and texture characteristics, each with strong regional features, thereby forming a diverse product series. These variations are closely associated with the composition of microbial communities and their metabolic activities (Wu Q. et al., 2021). A study has confirmed that microbial composition directly influences the safety, flavor, color, and texture of fermented products (Belleggia and Osimani, 2023). For instance, *lactic acid bacteria* promote the production of lactic and acetic acids, significantly contributing to product safety and flavor development (Zhao et al., 2014). Yeasts also play a crucial role in synthesizing aroma and taste compounds by decomposing fats and proteins (Padilla et al., 2014). However, certain microorganisms may produce undesirable metabolites; for example, specific species of *Pediococcus*, *Klebsiella*, and *Streptococcus* are known to form biogenic amines, which pose potential carcinogenic risks (Rabie et al., 2009). Therefore, analyzing the structure of microbial communities in fermented foods is of great significance for enhancing product quality and safety.

Current research on fermented fish products, both domestically and internationally, has primarily focused on the influence of starter cultures on flavor (Zeng et al., 2016b), changes in physicochemical properties (Zeng et al., 2013b), the dynamics of biogenic amines during fermentation (Zeng et al., 2013a), and analyses of microbial diversity (Zeng et al., 2016a). However, systematic studies examining the impact of regional differences on the microbial community structure and metabolic profiles of fermented fish remain unreported. Recent advances highlight that multi-omics approaches serve as powerful tools for deciphering the intricate relationships between microbial communities and flavor formation in fermented food (Shi et al., 2022). Unlike traditional single-method analyses, multi-omics enables systematic identification of key microorganisms and their metabolic contributions, providing new directions for precisely elucidating the roles of microbes in spontaneous fermentation systems. However, such comprehensive analyses remain largely unexplored for traditionally fermented fish from different geographical origins in Guizhou. Therefore, this study adopts a multi-omics strategy to address this gap.

Specifically, this study investigates fermented fish from Jinping, Liping, and Tianzhu Counties in Guizhou Province. Utilizing sensory evaluation, high-throughput sequencing, and untargeted metabolomics techniques, it systematically compares the quality characteristics, microbial diversity, and metabolite composition of fermented fish from these different regions. Furthermore, it establishes correlations between microbial communities and metabolite formation. This study aims to preliminarily elucidate the impact of microbial metabolism on the quality formation of fermented fish, providing scientific basis for optimizing traditional fermentation processes and enhancing product stability.

## 2 Materials and methods

### 2.1 Materials

The fermented fish samples were prepared using traditional local techniques in September 2018 and collected in March 2019. Samples were obtained from three representative locations in Qiandongnan Prefecture, Guizhou Province: Jinping County (“Shanxiang Flavor” specialty store), Liping County (RT-Mart supermarket specialty counter), and Tianzhu County (traditional handmade products from the farmers’ market). Three independent fermented fish specimens were collected from each location as biological replicates, totaling 9 samples, labeled as JP (Jinping), LP (Liping), and TZ (Tianzhu), respectively. After collection, all samples were immediately vacuum-packed and stored at 4 °C until subsequent analysis.

Under sterile conditions, surface-adhered materials (including superficial fish tissues, pepper, glutinous rice, and other fermentation substrates) were separately harvested from each fermented fish sample (JP, LP, TZ), thoroughly homogenized, and immediately flash-frozen in liquid nitrogen. The frozen samples were then stored at −80 °C for subsequent high-throughput sequencing and metabolite profiling analyses.

### 2.2 Physicochemical analysis of fermented fish

The pH values were measured with the pH meter (PH-100B, Shanghai Lichen Bangxi Instrument Technology Co., Ltd., Shanghai, China). Total acids (TA) were analyzed using a titration method with 0.10 M NaOH (Wu H. et al., 2021).

### 2.3 Sensory evaluations

Sensory evaluation was performed by a professional panel consisting of 5 trained assessors, all of whom possessed the ability to distinguish the five basic tastes, including sweetness, sourness, saltiness, bitterness, and umami. The evaluation indicators included four aspects: color, flavor, and texture. The experiment was conducted in a standard sensory laboratory with a constant indoor environment (temperature: 23 ± 2 °C; relative humidity: 55%). To minimize sensory fatigue and cross-contamination between samples, assessors were required to rinse their mouths with 100 mL of purified water between sample evaluations. Additionally, all assessors were prohibited from eating, drinking, or smoking within 1 h prior to the start of the evaluation.

### 2.4 High-throughput sequencing and sequence analyses

Genomic DNA was extracted from fermented fish samples of three experimental groups (three replicates per group) using the E. Z. N. A.® Soil DNA Kit (Omega Bio-tek, Norcross, GA, United States) according to the manufacturer’s instructions. The quality of the extracted DNA was verified by 2% agarose gel electrophoresis. The V3-V4 hypervariable regions of the bacterial 16S rRNA gene were amplified with primers 338F (5′-ACTCCTACGGGAGGCAGCAG-3′) and 806R (5′-GGACTACHVGGGTWTCTAAT-3′). For eukaryotes, the ITS region was amplified with primers ITS1F (5′-CTTGGTCATTTAGAGGAAGTAA-3′) and ITS2R (5′-GCTGCGTTCTTCATCGATGC-3′). The amplified products were quantified with a QuantiFluor™-ST fluorometer (Promega, Madison, WI, United States), and sequencing libraries were constructed from the purified PCR products using the TruSeq™ DNA Sample Prep Kit. Sequencing was performed on an Illumina platform (Shanghai Majorbio Bio-Pharm Technology Co., Ltd., Shanghai, China). The raw sequencing data have been deposited in the NCBI Sequence Read Archive (SRA) under BioProject accession numbers PRJNA1397516 (bacterial) and PRJNA1397525 (fungal).

### 2.5 Metabolite analysis

Liquid chromatography–mass spectrometry (LC–MS) analysis of the fermented fish was conducted using a Thermo Scientific UPLC-TripleTOF system coupled with a Fourier transform mass spectrometer (Shanghai Majorbio Bio-Pharm Technology Co., Ltd.). Chromatographic conditions were set as follows: The column temperature was maintained at 40 °C. A 10 μL aliquot of the sample was injected and separated on a BEH C18 column (100 mm × 2.1 mm i.d., 1.8 μm) prior to mass spectrometric detection. Mobile phase A consisted of water containing 0.1% formic acid, while mobile phase B was a mixture of acetonitrile and isopropanol (1:1, v/v) with 0.1% formic acid. The gradient elution program was optimized as follows: 0–3 min, mobile phase A linearly decreased from 95 to 80% with a corresponding linear increase of mobile phase B from 5 to 20%; 3–9 min, mobile phase A linearly reduced from 80 to 5%, and mobile phase B linearly increased from 20 to 95%; 9–13 min, both mobile phases A and B were maintained at 5 and 95%, respectively; 13.0–13.1 min, mobile phase A linearly rose from 5 to 95%, and mobile phase B linearly dropped from 95 to 5%; 13.1–16 min, mobile phases A and B were kept constant at 95 and 5%, respectively. Mass spectrometric conditions were configured as follows: Mass spectral signals of the sample were acquired in both positive and negative ion modes, with a mass scan range of m/z 50–1,000. The ion spray voltages were set at 5,000 V for positive ion mode and 4,000 V for negative ion mode. Other parameters included a declustering potential of 80 V, a curtain gas pressure of 30 psi, a nebulizer gas pressure of 50 psi, an auxiliary heating gas pressure of 50 psi, an ion source temperature of 500 °C, and a collision energy ranging from 20 to 60 V in cyclic mode.

### 2.6 Statistical analysis

All bioinformatics analyses of sequencing data were conducted on the Majorbio Cloud Platform.^1^ Specifically, the data for each sample were identified according to the index sequence and saved in FASTQ format. The fastp software^2^ was utilized for quality control of the paired-end raw sequencing data, while FLASH software^3^ was employed for splicing. The optimized sequences, following quality control and splicing, were denoised using DADA2 in Qiime2 to obtain amplicon sequence variants (ASVs). Taxonomic analysis of ASVs was performed using the classify-sklearn classifier in Qiime2, based on the Silva 16S rRNA gene database (version 138.2), with a confidence threshold of 0.7. For fungal ITS sequences, species annotation was carried out against the UNITE database (Release 10.0). Principal Coordinate Analysis (PCoA) based on the Bray-Curtis distance algorithm was employed to evaluate the similarity of microbial community structures among fermented fish samples. Additionally, Linear Discriminant Analysis Effect Size (LEfSe)^4^ was utilized to identify bacterial taxa with significantly different abundances from phylum to genus level between groups, applying the criteria of LDA > 4 and *p* < 0.05.

Raw metabolomic data were processed using Progenesis QI software (Waters Corporation, Milford, United States), resulting in a data matrix that includes retention time, mass-to-charge ratio (m/z), and peak intensity. The MS and MS/MS spectra were matched against public metabolic databases (HMDB^5^; Metlin^6^) as well as the in-house database of Majorbio. The mass error for MS was constrained to less than 10 ppm. Metabolites were identified based on the matching score of the secondary mass spectrum, and the resulting data matrix after database searching was uploaded to the Majorbio Cloud Platform (see Footnote 1) for further analysis. Supervised partial least squares discriminant analysis (PLS-DA) was conducted using the ropls package in R. Differential metabolites were identified based on the variable importance in projection (VIP) values derived from the PLS-DA model, with thresholds set at VIP > 1, *p* < 0.05, and fold change (FC) ≥ 1 or FC ≤ −1. KEGG pathway enrichment analysis and correlation analysis were performed using scipy.stats package in Python (Version 1.0.0).

## 3 Results

### 3.1 Physicochemical analysis of samples

As shown in Table 1, the pH values in the JP, LP, and TZ groups were 4.41 ± 0.01, 4.33 ± 0.02, and 4.20 ± 0.02, respectively; the TA values were 6.7 ± 0.03, 8.5 ± 0.02, and 14 ± 0.01, respectively.

**Table 1:** Physicochemical properties in fermentation.

Group	pH	TA (g lactic acid/L)
JP	4.46 ± 0.04^a^	6.74 ± 0.03^c^
LP	4.34 ± 0.01^b^	8.60 ± 0.05^b^
TZ	4.25 ± 0.04^b^	14.30 ± 0.12^a^

Data were reported as the mean value ± standard deviation of three replicates. Significant differences were observed between sample groups denoted by different letters (*p* < 0.05).

### 3.2 Sensory evaluation of each group of samples

Five trained food sensory evaluators were recruited for this study. A 100-point scale was adopted to quantitatively assess the color, appearance, flavor, and texture of fermented fish samples. The sensory evaluation results are presented in Table 2. Liping fermented fish achieved the highest overall score, exhibiting outstanding performance in multiple sensory attributes. Specifically, it featured rich and harmonious flavor, distinct yet pleasant sourness, natural ruddy color, and compact and elastic texture.

**Table 2:** Sensory evaluation results.

Group	Evaluation indicators			
	Color (30 points)	Flavor (40 points)	Texture (30 points)	Total score (100 points)
JP	Ruddy color	Normal flavor, pure sour taste	Soft fish meat	60
	15	25	20	
LP	Ruddy color	Rich flavor, strong sour taste	Compact fish meat	75
	15	35	25	
TZ	Light red color	Light flavor, weak sour taste	Mushy fish meat	50
	10	25	15	

### 3.3 Analysis of high-throughput sequencing results

#### 3.3.1 Differences in microbial diversity across samples

**Figure 1:**
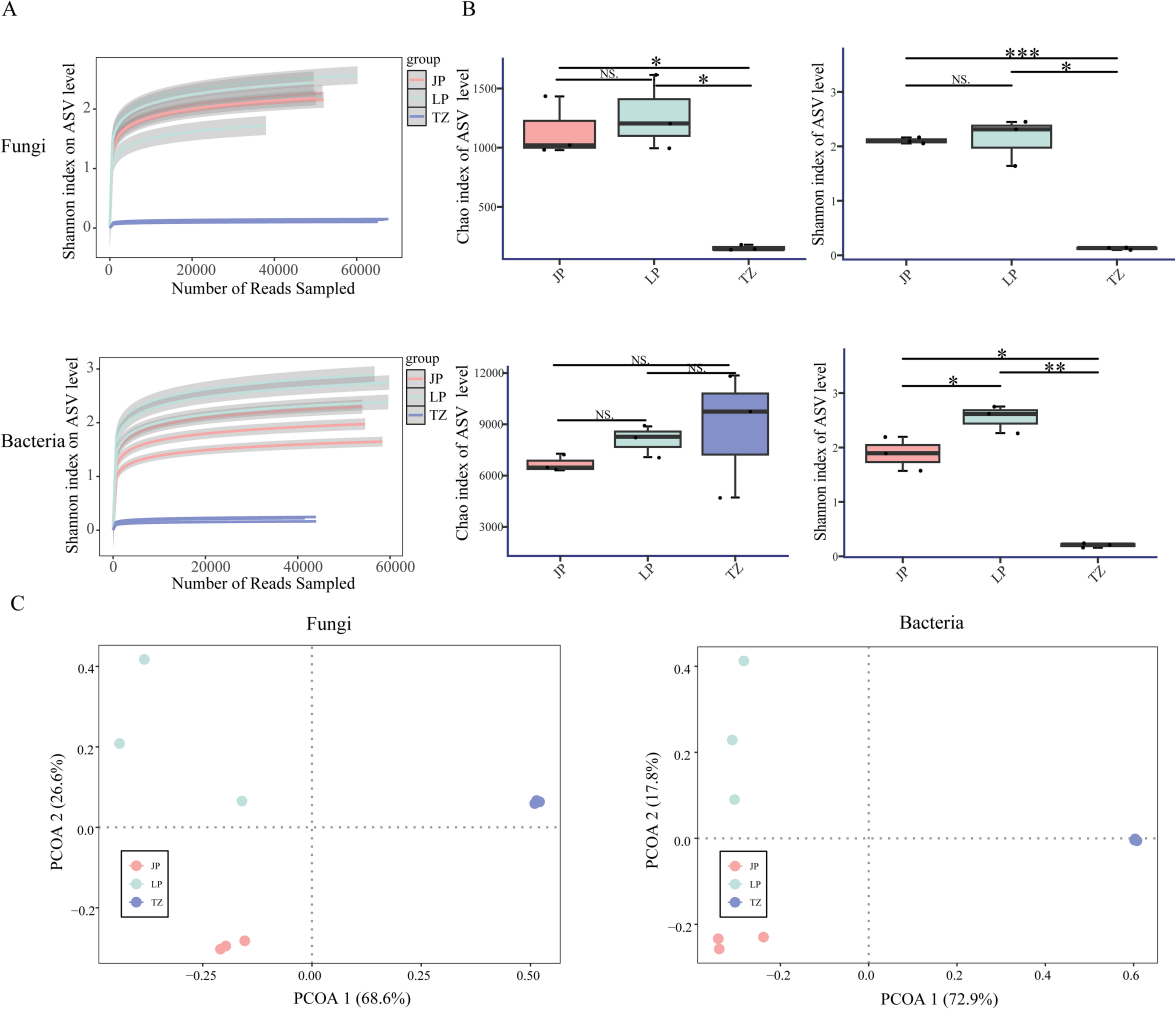
Microbial diversity of each group of samples (*n* = 3). After sequencing, fungal and bacterial rarefaction curves **(A)** and Chao and Shannon indices **(B)** were analyzed to assess α-diversity. PCoA **(C)** was performed to statistically evaluate microbial β-diversity levels among groups. *p < 0.05, **p < 0.01, and ***p < 0.001; NS, not significant.

The Illumina sequencing platform was employed for the microbial analysis of fermented fish samples. After data filtration and quality control, ITS and 16S rRNA amplicon sequencing generated 595,249 high-quality fungal sequences (totaling 172,077,743 bp, with an average length of 292 bp) and 472,324 bacterial sequences (totaling 199,542,123 bp, with an average length of 423 bp), as summarized in Supplementary Table S1. The coverage indices of both fungal and bacterial communities exceeded 99.9% across all samples, confirming that the sequencing data could comprehensively and accurately reflect the microbial diversity of the tested samples. The rarefaction curves of fungi and bacteria approached asymptotes (Figure 1A), further verifying that the sequencing depth was sufficient to capture the complete microbial diversity in the samples. To characterize the microbial diversity of fermented fish, fungal and bacterial community features were analyzed at the established sequencing depth (Figure 1B). In the fungal community, the LP group exhibited the highest species richness (Chao index = 1271.22) and community diversity (Shannon index = 2.14), while the TZ group showed the lowest values (Chao index = 153.05, Shannon index = 0.12) for both metrics among the three groups. Interestingly, in the bacterial community, although the TZ group had the highest Chao index (Chao index = 8775.03), its Shannon index (Shannon index = 0.20) was the lowest. Collectively, these results indicated that the TZ samples had the lowest microbial diversity in terms of both fungal and bacterial communities.

To evaluate the similarities and differences in community composition among the three fermented fish groups, principal coordinate analysis (PCoA) based on the Bray-Curtis distance was performed at the ASV level. The PCoA plot of the fungal community showed a clear separation among the JP, LP, and TZ groups (Figure 1C). For the bacterial community, samples from the JP and LP groups clustered closely together, and both groups were distinctly separated from the TZ group. These findings demonstrated significant differences in the fungal and bacterial community structures across the three fermented fish groups.

#### 3.3.2 Microbial composition of different samples

**Figure 2:**
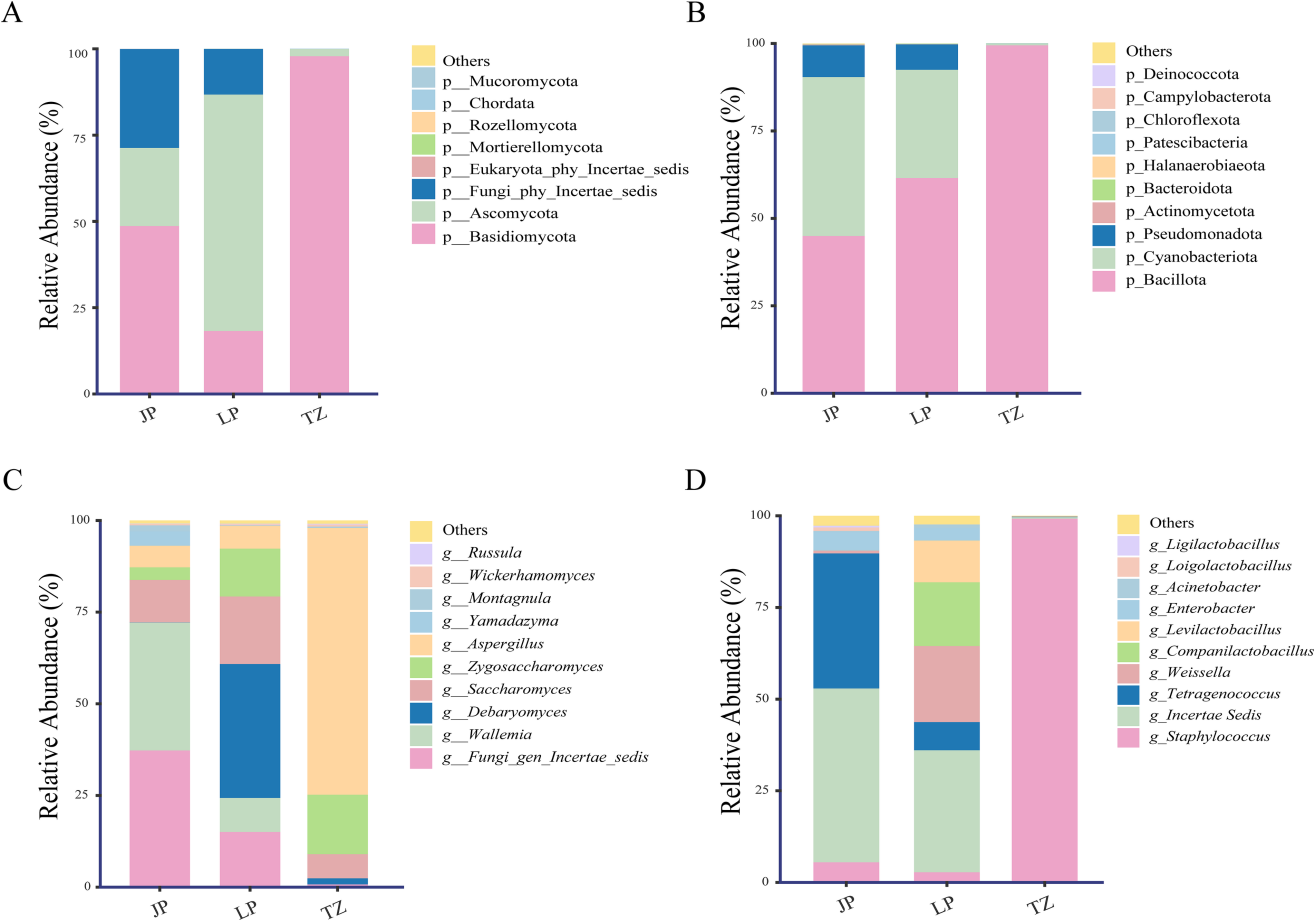
Microbial composition of each group of samples (*n* = 3). **(A)** Fungal phyla. **(B)** Bacterial phyla. **(C)** Fungal genera (mean relative abundance ≥ 1%). **(D)** Bacterial genera (mean relative abundance ≥ 1%).

To further explore the microbial community structure of samples from different groups, microbial compositions were classified at the phylum, class, order, family, and genus levels. The heterogeneity of microbial flora at the phylum and genus levels was analyzed accordingly. The composition of the fungal community is illustrated in Figure 2A. At the phylum level, Basidiomycota and Ascomycota collectively constituted the majority of the fungal community. Notably, their dominant positions exhibited significant variation across different sample groups. In the TZ group, Basidiomycota overwhelmingly dominated, with a relative abundance as high as 97.88%, whereas in the LP group, Ascomycota became the predominant phylum, accounting for 68.51%. The community structure of the JP group presents an intermediate pattern, with both Basidiomycota (48.68%) and Ascomycota (22.64%) forming its core components. The phylum-level distribution of bacteria in fermented fish is presented in Figure 2B. A total of three bacterial phyla were identified: Bacillota, Cyanobacteriota, and Pseudomonadota. Significant differences were observed in the relative abundances of Bacillota and Cyanobacteriota among the three sample groups. In the JP group, the levels of these two phyla were comparable; in contrast, the LP group exhibited approximately twice as many Bacillota as Cyanobacteriota. This trend became even more pronounced in the TZ group, where Bacillota overwhelmingly dominated, accounting for 99% of the community. Overall, the diversity of bacteria and fungi in fermented fish at the phylum level was relatively low.

At the genus level, significant differences were observed in the species composition and abundance of fungi among fermented fish from different production areas. Figure 2C presents the fungal genera with a relative abundance exceeding 1%. Distinct compositional patterns were identified among the three sample groups at the genus level. The JP group was primarily composed of *Fungi_gen_Incertae_sedis* (37.29%) and *Wallemia* (34.86%). The LP group displayed a more diverse assemblage, predominantly characterized by *Debaryomyces* (36.54%), followed by *Saccharomyces* (18.41%) and *Zygosaccharomyces* (13.04%). In contrast, the TZ group was overwhelmingly dominated by *Aspergillus* (72.80%), with *Zygosaccharomyces* (16.18%) as a secondary component. The genus-level composition of bacteria is illustrated in Figure 2D. In the JP group, the bacterial community was primarily composed of *Incertae Sedis* (47.41%) and *Tetragenococcus* (36.84%). Conversely, the LP group was characterized by the dominance of *Incertae Sedis* (33.29%) and *Weissella* (20.76%). Notably, in the TZ group, *Staphylococcus* (99.24%) nearly completely dominated, accounting for 99% of the relative abundance.

#### 3.3.3 Key biomarkers of different samples

**Figure 3:**
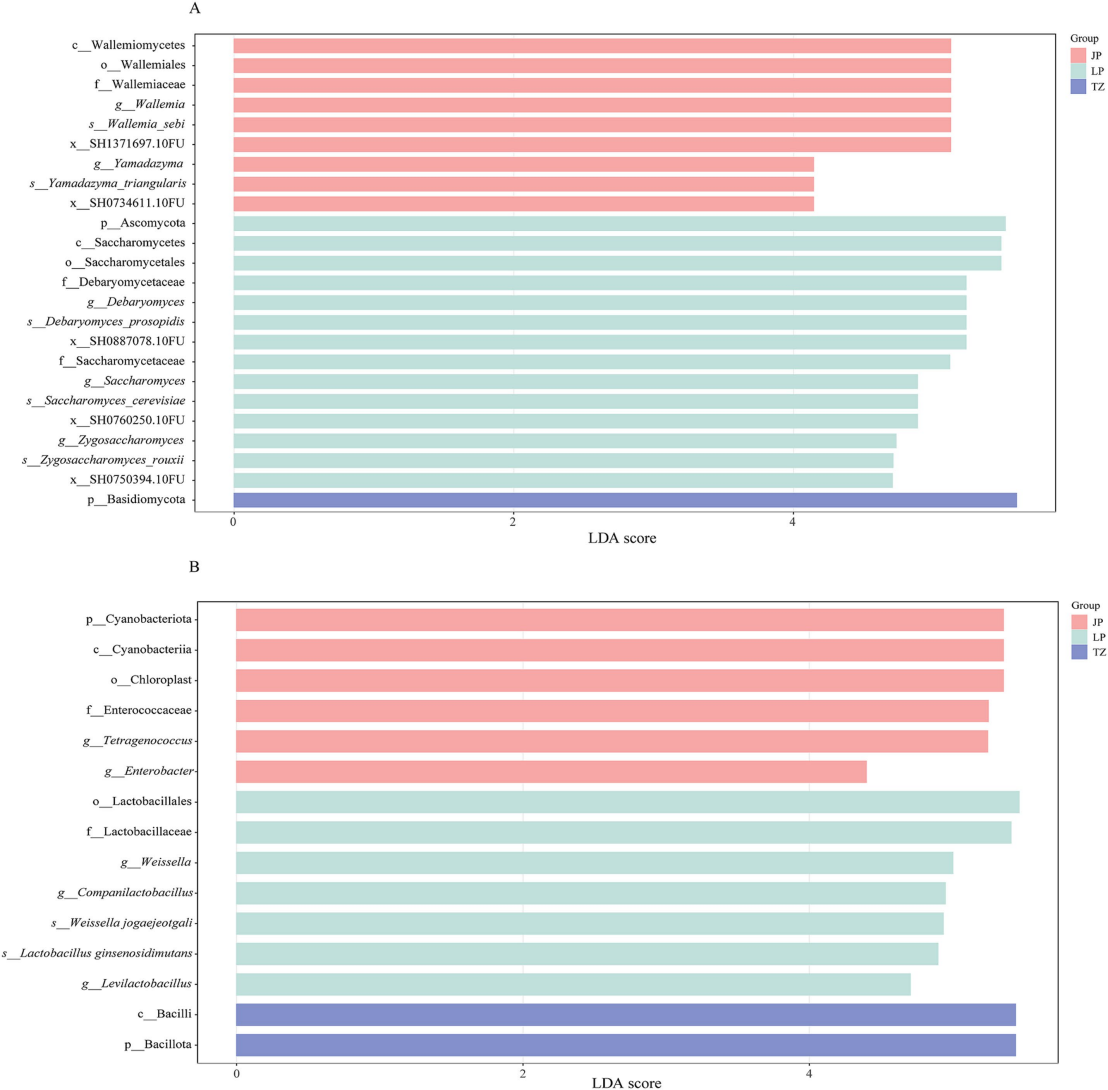
LEfSe analysis identified fungal **(A)** and bacterial **(B)** biomarkers. LDA scores were calculated for differential taxa (LDA > 4, *p* < 0.05).

To identify the key taxa underlying microbial community differences among groups, we conducted a LEfSe analysis to screen for genus-level biomarkers with intergroup discriminative capacity. The results revealed a total of five fungal genus-level biomarkers. The JP group exhibited 2 genus-level biomarkers, namely *Wallemia* and *Yamadazyma*, while the LP group displayed 3 genus-level biomarkers, including *Debaryomyces*, *Saccharomyces*, and *Zygosaccharomyces*. Additionally, five genus-level biomarkers were identified for bacteria. The JP group contained 2 genus-level biomarkers, *Tetragenococcus* and *Enterobacter*, whereas the LP group had 3 genus-level biomarkers: *Weissella*, *Companilactobacillus*, and *Levilactobacillus* (Figure 3).

### 3.4 Metabolite analysis

#### 3.4.1 Metabolite overview

**Figure 4:**
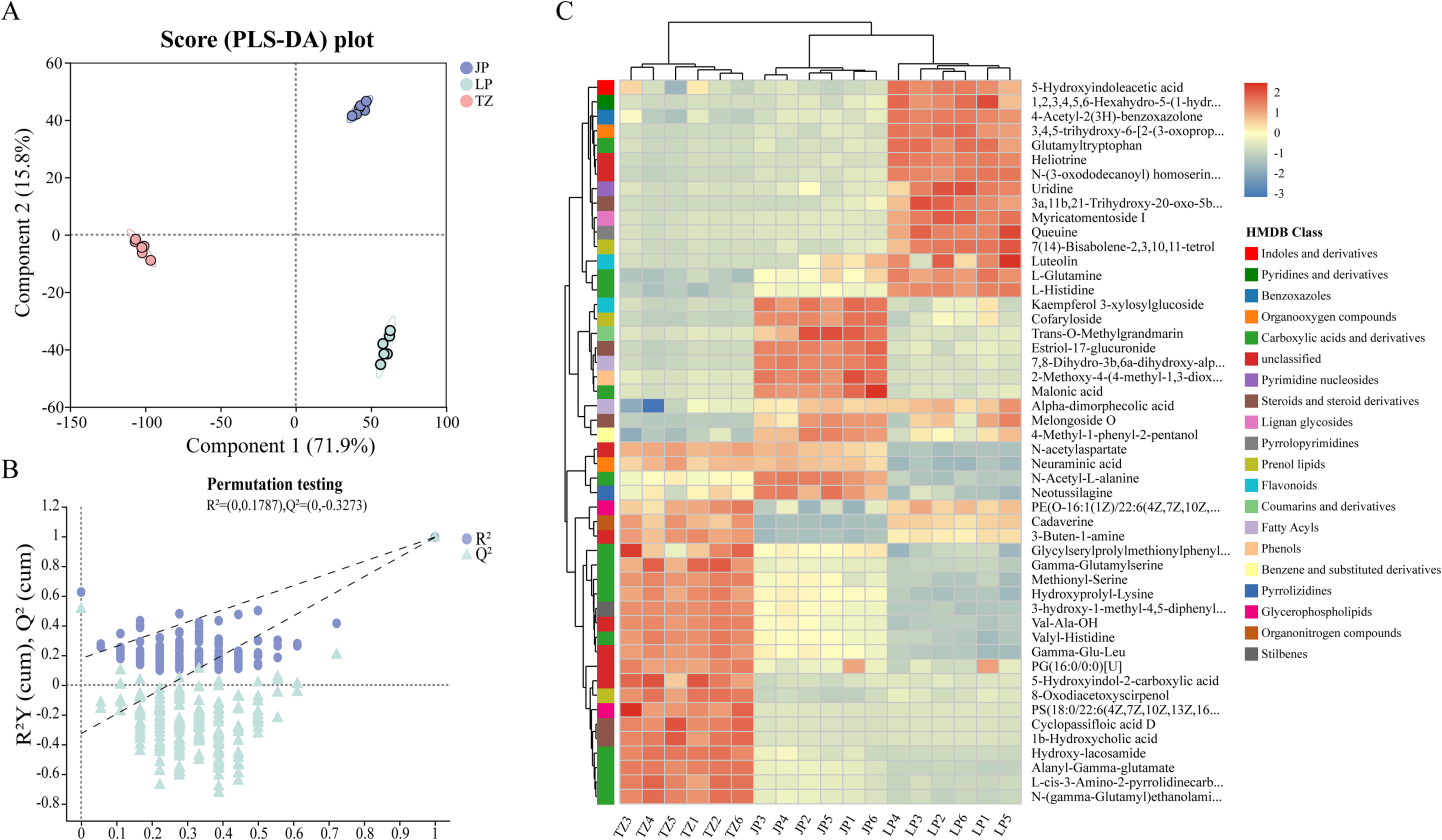
Metabolomic analysis of each group of samples (*n* = 6). Shown are partial least squares discriminant analysis (PLS-DA) of differential metabolites **(A)**, PLS-DA permutation test **(B)**, and cluster analysis of fermented fish samples **(C)**. Each row represents a metabolite, with colors ranging from red to blue indicating decreasing relative abundance.

A total of 1,372 metabolites were identified in this study via untargeted metabolomics analysis. As shown in Figure 4A, the partial least squares-discriminant analysis (PLS-DA) score plot revealed that the cumulative variance explained by the first principal component (PC1) and the second principal component (PC2) reached 87.70%, with PC1 accounting for 71.90% and PC2 for 15.80%. Samples from the three groups (JP, LP, and TZ) exhibited a clear separation trend in the score plot, indicating distinct differences in the metabolic profiles of fermented fish samples across different groups. To verify the validity of the PLS-DA model and prevent overfitting, a permutation test was conducted. The results demonstrated that the explanatory power (R^2^) of the model was consistently higher than its predictive power (Q^2^), and the intercept of the Q^2^ regression line with the y-axis was −0.3273. These results confirmed that the model had good fitting performance and reliable predictive ability and was suitable for the subsequent screening and analysis of differential metabolites (Figure 4B). This finding was further corroborated by the clustering heatmap (Figure 4C).

#### 3.4.2 Differential metabolites

**Figure 5:**
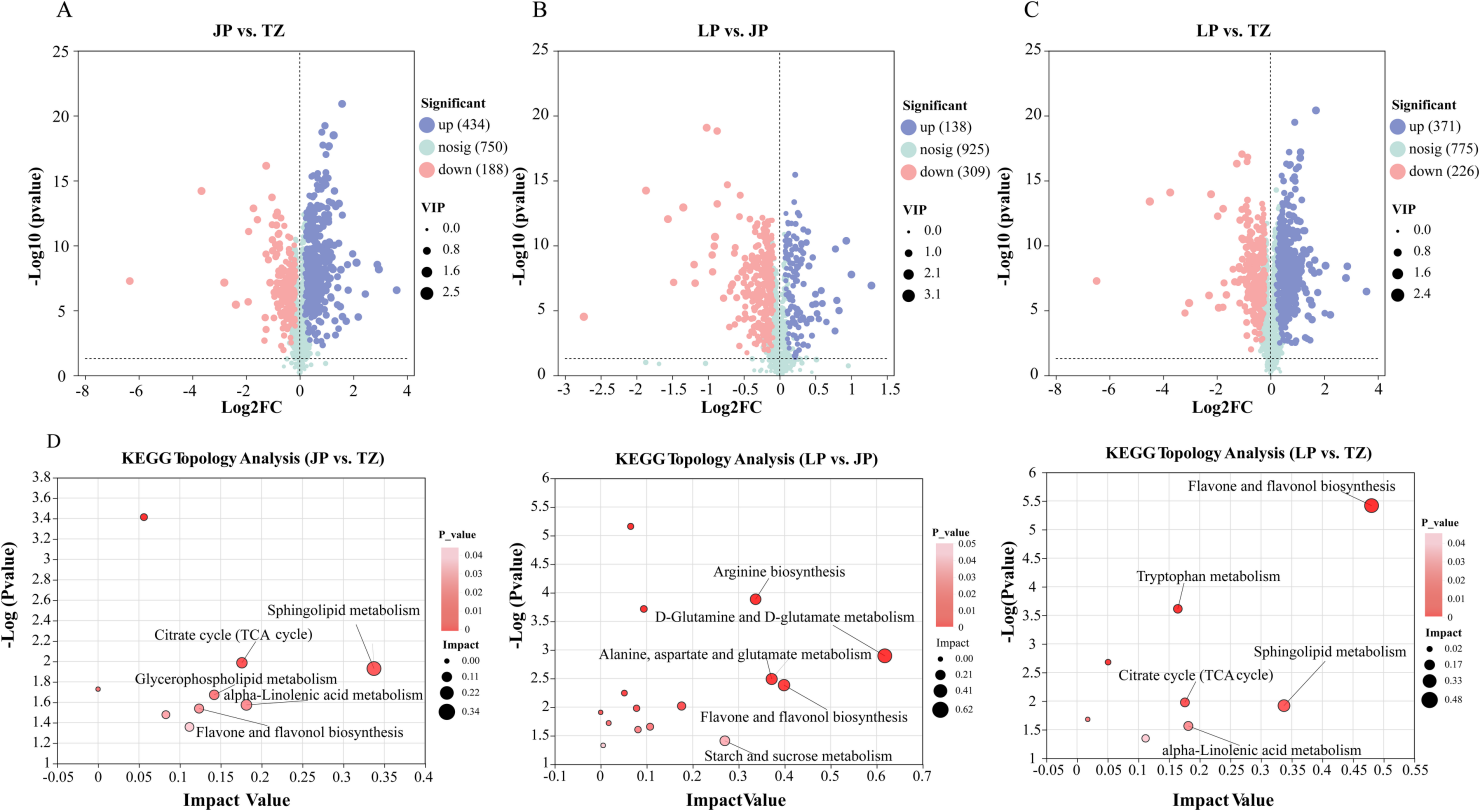
Volcano plot for significantly differentially expressed metabolites of **(A)** JP vs. TZ; **(B)** LP vs. JP; **(C)** LP vs. TZ. The enrichment of pathways. The X-axis represents the relative importance of metabolites in the pathway and the magnitude of the impact value **(D)**.

Based on the screening criteria (*p* < 0.05, |FC| ≥ 1), differentially expressed metabolites (DEMs) among multiple groups were identified. As shown in Figures 5A–C, a total of 622 DEMs were detected in the JP vs. TZ comparison group, including 434 upregulated and 188 downregulated ones. In the LP vs. JP group, 447 DEMs were identified, with 138 upregulated and 309 downregulated. For the LP vs. TZ group, 597 DEMs were obtained, consisting of 371 upregulated and 226 downregulated metabolites. To explore the potential biological functions associated with these DEMs, KEGG pathway enrichment analysis was further conducted on the groups (*p* < 0.05). The results indicated that DEMs in the JP vs. TZ group were significantly enriched in pathways such as sphingolipid metabolism, alpha-linolenic acid metabolism, the citrate cycle (TCA cycle), glycerophospholipid metabolism, and flavone and flavonol biosynthesis. DEMs in the LP vs. JP group were mainly related to D-glutamine and D-glutamate metabolism, flavone and flavonol biosynthesis, alanine, aspartate, and glutamate metabolism, and arginine biosynthesis, as well as starch and sucrose metabolism. For the LP vs. TZ group, the significantly enriched pathways included flavone and flavonol biosynthesis, sphingolipid metabolism, alpha-linolenic acid metabolism, the citrate cycle (TCA cycle), and tryptophan metabolism (Figure 5D).

### 3.5 Correlation between microorganisms and metabolites in different samples

**Figure 6:**
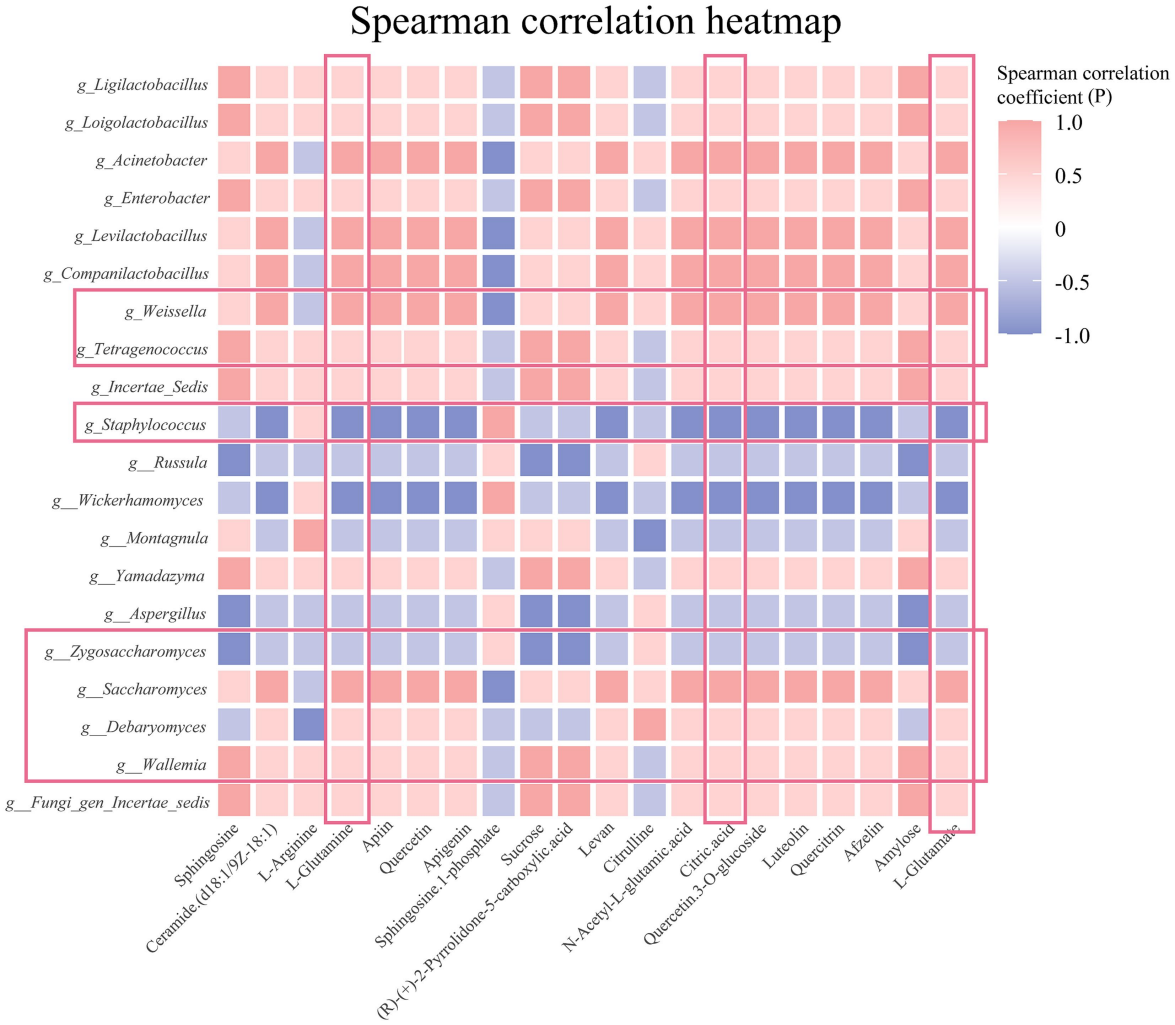
The correlation between microbiota and differential metabolites in each group of samples. Based on Spearman correlation coefficients, a correlation analysis was performed between the top 10 fungal and bacterial genera by relative abundance and the 20 selected DEMs.

Spearman correlation analysis was employed to investigate the associations between microbes and metabolites in three groups of fermented fish samples. As shown in Figure 6, *Weissella*, *Saccharomyces*, and *Debaryomyces* exhibited positive correlations with L-glutamine, citric acid, and L-glutamate, while showing negative correlations with L-arginine and sphingosine 1-phosphate. In contrast, *Staphylococcus* displayed the opposite correlation pattern with the metabolites essentially.

## 4 Discussion

This study systematically investigates the quality differences and underlying microbial and metabolic mechanisms of fermented fish from different producing areas (JP, LP, and TZ) by integrating sensory evaluation, untargeted metabolomics, and microbiomics. The results reveal significant variations in sensory quality, metabolic profiles, and microbial community structures among the three groups. Notably, the LP group achieved the highest comprehensive sensory score, characterized by a harmonious flavor, distinct sourness, and firm texture. The JP group ranked second, while the TZ group had the lowest score. Further analysis indicates a significant correlation between the unique microbial community structure of each group and differential metabolites, suggesting that microbial metabolic activity is a key driver of fermented fish quality formation.

Microbial analysis revealed that the TZ group exhibited the lowest α-diversity among both fungi and bacteria, with *Staphylococcus* accounting for over 99% of the relative abundance, indicating a highly simplified community structure. Although reduced microbial diversity may sometimes enhance fermentation stability, the absolute dominance of a single genus could lead to simplified metabolic functions, hindering the diversified production of flavor metabolites and consequently reducing sensory quality (Ji et al., 2022). This is likely the primary reason for the low sensory scores in the TZ group. Additionally, the singular dominance of *Staphylococcus* poses potential safety risks (Zhang et al., 2025). Certain *Staphylococcus* strains can produce biogenic amines such as tyramine and histamine, whose excessive accumulation not only generates bitter or chemical off-flavors but may also cause adverse reactions, including headaches and abnormal blood pressure (Atasoy et al., 2024). In highly homogeneous microbial communities, the absence of other microorganisms for biodegradation or metabolic competition of biogenic amines makes them more prone to accumulation (Hiruma et al., 2020; Wang et al., 2024). In contrast, the JP and LP groups exhibited a more diverse genus composition, including *Weissella*, *Saccharomyces*, and *Debaryomyces*. These genera are typically associated with flavor formation in traditional fermented foods (Xie et al., 2026; Xu et al., 2023). The superior sensory characteristics of the LP group were closely linked to its unique microbial community structure. As a common probiotic, *Weissella* participates in glycolysis and lactic acid fermentation, producing lactic acid to lower pH, inhibit spoilage bacteria, enhance storage stability, and impart a mild sour taste (Wan et al., 2023; Zhao et al., 2024). Simultaneously, it converts L-glutamine into L-glutamic acid through glutaminase activity, enhancing product umami (Li et al., 2009). In the LP group, the abundance of *Saccharomyces* was significantly higher than in the other groups, which could regulate system acidity, promote the accumulation of flavor compounds, and form interactive relationships with lactic acid bacteria, further optimizing flavor harmony (Wenkang et al., 2024). Meanwhile, *Saccharomyces* can also generate esters, higher alcohols, and other compounds through amino acid decomposition and lipid degradation, imparting rich and complex aromas to fermented fish products (Bai et al., 2025). Metabolomic analysis revealed significant differences in metabolite composition among the three groups, with the differential metabolites primarily enriched in lipid metabolism, amino acid metabolism, and flavonoid biosynthesis pathways. The LP group exhibited more pronounced enrichment in amino acid metabolism (particularly alanine, aspartate, and glutamate metabolism) and flavonoid biosynthesis pathways, with significantly upregulated levels of L-glutamine, L-glutamate, and citric acid. Notably, citric acid contributes fresh acidity, regulates pH, and participates in microbial energy metabolism (Mao et al., 2023); sucrose can balance salty and umami flavors, and also serves as a substrate for Maillard reaction and caramelization to form complex aromas and colors (Tamanna and Mahmood, 2015). KEGG enrichment analysis further validated the above conclusions. The differential metabolites in the LP group were significantly enriched in pathways such as alanine, aspartate, and glutamate metabolism, corresponding to the conversion of glutamine to glutamate and citrate generation processes. In contrast, the TZ group showed no such enrichment, indicating its deficiency in key taste substance biosynthesis pathways. During the fermentation process, microbial diversity showed a positive correlation with metabolic diversity (Chen et al., 2023; Wu et al., 2025). In the LP group, key microorganisms such as *Weissella* and *Saccharomyces* exhibited significant positive correlations with the aforementioned flavor metabolites. Furthermore, microbial interactions serve as the core driver of fermented food quality (Ji et al., 2025). Co-fermentation of *Saccharomyces cerevisiae* with *lactic acid bacteria* can enhance amino acid-derived flavors and reduce biogenic amine accumulation (Pang et al., 2023; Wenkang et al., 2023). Thus, we hypothesize that the coexistence of *Weissella* and *Saccharomyces* in the LP group may form a synergistic metabolic network, promoting the TCA cycle and glutamine-glutamate conversion, thereby improving product flavor and umami characteristics. However, the high-throughput sequencing technology employed in this study has inherent limitations that preclude in-depth analysis at the species level. Furthermore, we did not detect key safety indicators such as biogenic amines, histamine, and potential pathogenic bacteria, which serve as crucial criteria for comprehensive quality assessment of traditional fermented fish products. In future work, we will employ metagenomic sequencing technology to conduct more systematic research, incorporating the aforementioned safety indicators to more comprehensively analyze the risks and benefits of different microbial communities.

In conclusion, this study systematically elucidates for the first time the microbial and molecular basis of flavor quality differences in fermented fish from different production areas of Guizhou through multi-omics integration analysis. The LP group demonstrated superior performance in flavor, acidity, and texture, attributed to its more balanced microbial diversity and the presence of characteristic functional genera such as *Weissella* and *Saccharomyces*. This study not only highlights the pivotal role of specific microorganisms in the flavor development of fermented fish but also offers a theoretical foundation for the targeted regulation of microbial communities aimed at enhancing product quality. Future research could focus on constructing synthetic microbial communities or conducting multi-omics integration analyses to further investigate the regulatory networks between key strains and metabolic pathways, thereby providing scientific support for the standardized production and quality enhancement of traditional fermented fish.

## 5 Conclusion

This study represents the first systematic comparison of the metabolomic and microbiomic profiles of fermented fish samples from the JP, LP, and TZ regions. Key microbial biomarkers, including *Weissella*, *Saccharomyces*, and *Staphylococcus*, were identified, alongside characteristic metabolites such as L-glutamine, citric acid, and L-glutamate. Correlation analysis further elucidated the potential roles of these microorganisms in flavor formation and the accumulation of safety-related metabolites. This research provides a scientific foundation for geographical traceability and the development of specialized starters for fermented fish.

## Data Availability

The datasets presented in this study can be found in online repositories. The names of the repository/repositories and accession number(s) can be found below: https://www.ncbi.nlm.nih.gov/, PRJNA1397516; https://www.ncbi.nlm.nih.gov/, PRJNA1397525.

## Footnotes

1
https://cloud.majorbio.com

2
https://github.com/OpenGene/fastp

3
http://www.cbcb.umd.edu/software/flash

4
http://huttenhower.sph.harvard.edu/LEfSe

5
http://www.hmdb.ca/

6
https://metlin.scripps.edu/

## References

[ref1] Atasoy M. Álvarez O. A. Cenian A. Djukić-Vuković A. Lund P. A. Ozogul F. . (2024). Exploitation of microbial activities at low pH to enhance planetary health. FEMS Microbiol. Rev. 48:fuad062. doi: 10.1093/femsre/fuad062, 37985709 PMC10963064

[ref2] Bai C. Fan B. Hao J. Yao Y. Ran S. Wang H. . (2025). Changes in microbial community diversity and the formation mechanism of flavor metabolites in industrial-scale spontaneous fermentation of cabernet sauvignon wines. Foods (Basel, Switzerland) 14:235. doi: 10.3390/foods14020235, 39856901 PMC11764576

[ref3] Belleggia L. Osimani A. (2023). Fermented fish and fermented fish-based products, an ever-growing source of microbial diversity: a literature review. Food Res. Int. (Ottawa) 172:113112. doi: 10.1016/j.foodres.2023.113112, 37689879

[ref4] Chen G. Yuan Y. Tang S. Yang Z. Wu Q. Liang Z. . (2023). Comparative analysis of microbial communities and volatile flavor components in the brewing of Hongqu rice wines fermented with different starters. Curr. Res. Food Sci. 7:100628. doi: 10.1016/j.crfs.2023.100628, 38021257 PMC10660030

[ref7] Hiruma S. Ishihara M. Nakamura S. Sato Y. Asahina H. Fukuda K. . (2020). Bioshell calcium oxide-containing liquids as a sanitizer for the reduction of histamine production in raw Japanese pilchard, Japanese horse mackerel, and chub mackerel. Foods (Basel, Switzerland) 9:964. doi: 10.3390/foods9070964, 32708249 PMC7404465

[ref8] Ji J. Jiang X. Song P. Yang Q. Sun M. Dong Z. . (2025). Multi-omics insights into microbial interactions and fermented food quality. Microorganisms 13:2679. doi: 10.3390/microorganisms13122679, 41471883 PMC12734765

[ref9] Ji X. Yu X. Wu Q. Xu Y. (2022). Initial fungal diversity impacts flavor compounds formation in the spontaneous fermentation of Chinese liquor. Food Res. Int. (Ottawa) 155:110995. doi: 10.1016/j.foodres.2022.110995, 35400416

[ref10] Li J. Izumimoto M. Yonehara M. Hirotsu S. Kuriki T. Naito I. . (2009). The influence of fig proteases on the inhibition of angiotensin I-converting and GABA formation in meat. Anim. Sci. J. 80, 691–696. doi: 10.1111/j.1740-0929.2009.00682.x, 20163660

[ref11] Mao X. Yue S. J. Xu D. Q. Fu R. J. Han J. Z. Zhou H. M. . (2023). Research Progress on flavor and quality of Chinese Rice wine in the brewing process. ACS Omega 8, 32311–32330. doi: 10.1021/acsomega.3c04732, 37720734 PMC10500577

[ref12] Padilla B. Belloch C. López-Díez J. J. Flores M. Manzanares P. (2014). Potential impact of dairy yeasts on the typical flavour of traditional ewes' and goats' cheeses. Int. Dairy J. 35, 122–129. doi: 10.1016/j.idairyj.2013.11.002

[ref13] Pang Z. Hao J. Li W. Du B. Guo C. Li X. . (2023). Investigation into spatial profile of microbial community dynamics and flavor metabolites during the bioaugmented solid-state fermentation of baijiu. Food Biosci. 56:103292. doi: 10.1016/j.fbio.2023.103292

[ref14] Rabie M. Simon-Sarkadi L. Siliha H. El-seedy S. El Badawy A.-A. (2009). Changes in free amino acids and biogenic amines of Egyptian salted-fermented fish (Feseekh) during ripening and storage. Food Chem. 115, 635–638. doi: 10.1016/j.foodchem.2008.12.077

[ref15] Shi H. An F. Lin H. Li M. Wu J. Wu R. (2022). Advances in fermented foods revealed by multi-omics: a new direction toward precisely clarifying the roles of microorganisms. Front. Microbiol. 13:1044820. doi: 10.3389/fmicb.2022.1044820, 36590428 PMC9794733

[ref16] Sun H. Liu X. Wang L. Sang Y. Sun J. (2022). Exploring the fungal community and its correlation with the physicochemical properties of Chinese traditional fermented fish (Suanyu). Foods (Basel, Switzerland) 11:1721. doi: 10.3390/foods11121721, 35741919 PMC9222310

[ref17] Tamanna N. Mahmood N. (2015). Food processing and Maillard reaction products: effect on human health and nutrition. Int. J. Food Sci. 2015:526762. doi: 10.1155/2015/526762, 26904661 PMC4745522

[ref19] Wan X. Takala T. M. Huynh V. A. Ahonen S. L. Paulin L. Björkroth J. . (2023). Comparative genomics of 40 *Weissella paramesenteroides* strains. Front. Microbiol. 14:1128028. doi: 10.3389/fmicb.2023.1128028, 37065164 PMC10102382

[ref20] Wang X. Liu X. Sun C. Cheng Y. Li Z. Qiu S. . (2024). Effect of temperature on the quality and microbial community during Daocai fermentation. Food Chem. X 24:101827. doi: 10.1016/j.fochx.2024.101827, 39421152 PMC11483281

[ref21] Wenkang H. Fuyi H. Hongyan C. Jiamin L. Rui Z. Qin C. . (2024). Influence of acid-reducing *Saccharomyces cerevisiae* on the microbial communities and metabolites of Suanyu. Food Res. Int. (Ottawa) 181:114117. doi: 10.1016/j.foodres.2024.114117, 38448112

[ref22] Wenkang H. Jingui L. Wei Z. Jiangli W. Zhengbin Y. Furong Z. . (2023). Multi-omics analysis reveals the microbial interactions of S. Cerevisiae and *L. plantarum* on Suanyu, Chinese traditional fermented fish. Food Res. Int. 174:113525. doi: 10.1016/j.foodres.2023.113525, 37986426

[ref23] Wu H. Dong J.-J. Dai Y.-Q. Liu X.-L. Zhou J.-Z. Xia X.-D. (2021). Effects of lactic acid bacteria fermented yellow whey on the protein coagulation and isoflavones distribution in soymilk. Food Chem. 334:127484. doi: 10.1016/j.foodchem.2020.127484, 32711263

[ref24] Wu J. Lin N. Yang J. Zhang X. Wu K. You X. . (2025). Influence of inoculating Saccharomyces cerevisiae and Levilactobacillus brevis on the quality of fermented large yellow croaker (*Larimichthys crocea*): biogenic amines, volatile components, and microbial communities changes. Foods (Basel, Switzerland) 14:3690. doi: 10.3390/foods14213690, 41227661 PMC12607568

[ref25] Wu Q. Zhu Y. Fang C. Wijffels R. H. Xu Y. (2021). Can we control microbiota in spontaneous food fermentation? – Chinese liquor as a case example. Trends Food Sci. Technol. 110, 321–331. doi: 10.1016/j.tifs.2021.02.011

[ref26] Xie S. Wang R. Zhong W. Qin L. Zhang W. Chen Q. . (2026). Analysis of fermentation characteristics, nutritional and flavor profiles of Xuxiang kiwifruit juice fermented by different lactic acid bacterial strains, and changes in antioxidant activity after *in vitro* digestion. J. Sci. Food Agric. 106, 904–918. doi: 10.1002/jsfa.70218, 41074744

[ref27] Xu Y. Qiao X. He L. Wan W. Xu Z. Shu X. . (2023). Airborne microbes in five important regions of Chinese traditional distilled liquor (baijiu) brewing: regional and seasonal variations. Front. Microbiol. 14:1324722. doi: 10.3389/fmicb.2023.1324722, 38264484 PMC10803494

[ref28] Zeng X. Chen X. Zhang W. (2016a). Characterization of the microbial Flora from Suan Yu, a Chinese traditional low-salt fermented fish. J. Food Process. Preservat. 40, 1093–1103. doi: 10.1111/jfpp.12690

[ref29] Zeng X. Xia W. Jiang Q. Yang F. (2013b). Chemical and microbial properties of Chinese traditional low-salt fermented whole fish product Suan yu. Food Control 30, 590–595. doi: 10.1016/j.foodcont.2012.07.037

[ref30] Zeng X. Xia W. Yang F. Jiang Q. (2013a). Changes of biogenic amines in Chinese low-salt fermented fish pieces (Suan yu) inoculated with mixed starter cultures. Int. J. Food Sci. Technol. 48, 685–692. doi: 10.1111/ijfs.12010

[ref31] Zeng X. Zhang W. Zhu Q. (2016b). Effect of starter cultures on the quality of Suan yu, a Chinese traditional fermented freshwater fish. Int. J. Food Sci. Technol. 51, 1774–1786. doi: 10.1111/ijfs.13140

[ref32] Zhang X. Wang F. Zhang M. Xu H. Huang Y. (2025). Safety evaluation of Staphylococcus and its application in soy sauce brewing. Food Sci. Technol. Int. 31, 454–465. doi: 10.1177/10820132231182459, 38105439

[ref33] Zhang Q. Zhao F. Shi T. Xiong Z. Gao R. Yuan L. (2023). Suanyu fermentation strains screening, process optimization and the effect of thermal processing methods on its flavor. Food Res. Int. (Ottawa) 173:113296. doi: 10.1016/j.foodres.2023.113296, 37803608

[ref34] Zhao Y. W. Wu Z. F. Shen X. Q. Weng P. F. Chen J. J. (2014). Bacteria community analysis by quantitative real-time PCR of fermenting wax gourd and its changes of organic acids. J. Food Process. Preservat. 38, 1653–1659. doi: 10.1111/jfpp.12127

[ref35] Zhao J. Zhao J. Zang J. Peng C. Li Z. Zhang P. (2024). Isolation, identification, and evaluation of lactic acid bacteria with probiotic potential from traditional fermented sour meat. Front. Microbiol. 15:1421285. doi: 10.3389/fmicb.2024.1421285, 39726969 PMC11669687

