# Supplementary material for: Multi-omics insights into the microbial and metabolic drivers of regional flavor diversity in Guizhou traditional fermented fish
Source: Front Microbiol. 2026 Jun 18;17:1821104. doi: 10.3389/fmicb.2026.1821104 (PMC13323630; doi:10.3389/fmicb.2026.1821104)
Supplement: Supplementary file 1 [file Table_1.DOCX]

Supplementary Material

**Multi-Omics Insights into the Microbial and Metabolic Drivers of Regional Flavor Diversity in Guizhou Traditional Fermented Fish**

**Anqin Zhu^1^, YanLi Wang^2^, Jin Zhang^1^, Shiping Lu^3^, Chuanbo Zhang^1*^**

^1^Laboratory of Microbial Resources and Industrial Application, College of Life Sciences, Guizhou Normal University, Guiyang, 550025, China

^2^ Department of Agronomic Engineering, Guizhou Vocational College of Agriculture, Guizhou 551400, China

^3^ Huangping Yedonghe Yuan Ecological Recycling Farming Co., Ltd.

^*^Corresponding Author: Chuanbo Zhang ([zhangchuanbo2004@163.com](mailto:zhangchuanbo2004@163.com))

The supporting information (a total of 1 page, including the cover sheet) contains assays for 1 Supplementary table (Table 1).

## Supplementary Table

**Supplementary Table 1.** Based on 16S rRNA and ITS augmented sequencing, the samples after sequencing result

| Sample | Fungi | | | |  | | | Bacteria | | |  | |
| --- | --- | --- | --- | --- | --- | --- | --- | --- | --- | --- | --- | --- |
|  | Seqence | Base/bp | Mean_length/bp | coverage | | Seqence | Base/bp | | Mean_length/bp | | | coverage |
| JP_1 | 73019 | 20686614 | 283.30454 | 0.999587 | | 58351 | 24460739 | | | 419.2 | | 0.9992 |
| JP_2 | 73065 | 20912907 | 286.22332 | 0.999525 | | 53986 | 22620158 | | | 419.00044 | | 0.99907 |
| JP_3 | 69814 | 19703109 | 282.22289 | 0.99938 | | 54586 | 22727393 | | | 416.35938 | | 0.99925 |
| LP_1 | 72828 | 20798556 | 285.58461 | 0.999476 | | 56624 | 23905401 | | | 422.17789 | | 0.99907 |
| LP_2 | 44543 | 16006012 | 359.33844 | 0.999495 | | 59798 | 25166343 | | | 420.85593 | | 0.99923 |
| LP_3 | 62357 | 19474898 | 312.31294 | 0.999685 | | 59595 | 25170473 | | | 422.35881 | | 0.99914 |
| TZ_1 | 65157 | 17808557 | 273.31763 | 0.999584 | | 43913 | 18834306 | | | 428.90046 | | 0.9998 |
| TZ_2 | 66751 | 18227764 | 273.07103 | 0.999793 | | 43987 | 18868844 | | | 428.9641 | | 0.99973 |
| TZ_3 | 67715 | 18459326 | 272.6032 | 0.999633 | | 41484 | 17788466 | | | 428.80306 | | 0.99973 |
